# An Assessment of the Correlation Between Tooth Size and Agenesis of Maxillary Lateral Incisor in Subjects Undergoing Orthodontic Treatment

**DOI:** 10.7759/cureus.25642

**Published:** 2022-06-03

**Authors:** Manish S Dagdiya, Lalima Kumari, Amruta Narendra Motarwar, Richashree ., Kumar Anand, Satya Prakash

**Affiliations:** 1 Department of Orthodontics and Dentofacial Orthopaedics, Saraswati-Dhanwantari Dental College and Hospital, Parbhani, IND; 2 Department of Orthodontics and Dentofacial Orthopaedics, Patna Dental College and Hospital, Patna, IND; 3 Department Of Orthodontics and Dentofacial Orthopaedics, Saraswati-Dhanwantari Dental College and Hospital, Parbhani, IND; 4 Department of Orthodontics and Dentofacial Orthopaedics, Buddha Institute of Dental Sciences and Hospital, Patna, IND; 5 Department of Oral Medicine and Radiology, Buddha Institute of Dental Sciences and Hospital, Patna, IND; 6 Department of Oral Medicine and Radiology, Dr B. R. Ambedkar Institute of Dental Sciences and Hospital, Patna, IND

**Keywords:** tooth size., orthodontic treatment, maxillary lateral incisor, missing teeth, agenesis

## Abstract

Background and objective

Occlusion rehabilitation and restoration are difficult in subjects with congenitally missing lateral incisors, either unilaterally or bilaterally, and often lead to malocclusion and warrant replacement. The present study was conducted to assess the agenesis of maxillary lateral incisor unilaterally and bilaterally and to examine tooth size discrepancy in agenesis subjects undergoing orthodontic treatment.

Materials and methods

We assessed 32 dental casts of both genders (17 males and 15 females) with missing maxillary lateral incisors either unilaterally or bilaterally. Mesiodistal dimensions were measured and a comparison of tooth sizes was done for control and test groups. The data were assessed and the results were documented.

Results

Lateral incisors of the maxillary arch were statistically smaller in the test group compared to the control group. This was true for both males and females (p=0.001 for both). A similar finding was observed with respect to the overall study group (p<0.0001).

Conclusion

Based on our findings, maxillary lateral incisor agenesis plays a role in malocclusion development.

## Introduction

Occlusion rehabilitation and restoration can be challenging in subjects with congenitally missing lateral incisors, either unilaterally or bilaterally. These occlusion rehabilitation problems are usually encountered by general dentists, orthodontists, and prosthodontists. The treatment choices for congenitally missing lateral incisors are mesial canine movement, fixed dental prosthesis, and implant-supported prosthesis [1].

To assess the space needed for restoration and the size of the missing lateral incisor, the lateral incisor of the contralateral side is usually used as a guide. However, a limitation of this practice is the absence or peg shape of contralateral lateral incisors in subjects with congenitally missing lateral incisors. In such cases, to determine the missing tooth size, two other methods are widely accepted and used. They include golden proportion where 62% width of the central incisor comprises the missing lateral incisor and Bolton analysis to assess the space needed for replacing the missing lateral incisor [2].

Tooth and jaw size discrepancy in both maxillary and mandibular arch can make a space of 6-7 mm insufficient for replacing the missing lateral incisor with the implant. This is mostly seen in cases with coincident midlines, ideal horizontal and vertical overlaps, and class I canine relationships [3]. Mesiodistal width of maxillary lateral incisor was found to be the most significant tooth contributing to arch size-tooth size discrepancy in comparison to buccolingual dimension as described in the published literature [4].

Genetic factors including mutations of PAX9 and MSX1 genes may lead to tooth size discrepancies and tooth agenesis. The mutation of the gene PAX9 can also lead to the formation of teeth smaller than normal. Missing lateral incisor is more commonly seen in women compared to men. Higher tooth size is usually reported in men [5].

The relationship between tooth agenesis and tooth size is not extensively studied, and data related to this is scarce in the literature. Recent data suggest that less space is needed for placing newer dental implants, thereby reducing the tooth size needed for placement. In light of this, the present study was conducted to assess the agenesis of maxillary lateral incisor unilaterally and bilaterally and to examine tooth size discrepancy in agenesis subjects undergoing orthodontic treatment.

## Materials and methods

Study design and setting

This was an observational clinical study. The study was conducted after obtaining approval from the concerned Ethical Committee (ethical approval no: ICMCH/2020/19). The study was carried out on dental casts collected from the Department of Orthodontics and Dentofacial Orthopedics. A total of 32 casts were included from both genders. There were 17 male casts and 15 female casts in the study.

All casts had unilateral/bilateral missing maxillary lateral incisors. There were 19 unilateral missing lateral incisors and 13 bilateral missing lateral incisors. The age of study subjects ranged from 11 to 42 years with a mean age of 15.78 years. For the purpose of comparison, 32 controls matched for age and sex were also included and compared against tests with missing lateral incisors.

**Figure 1:**
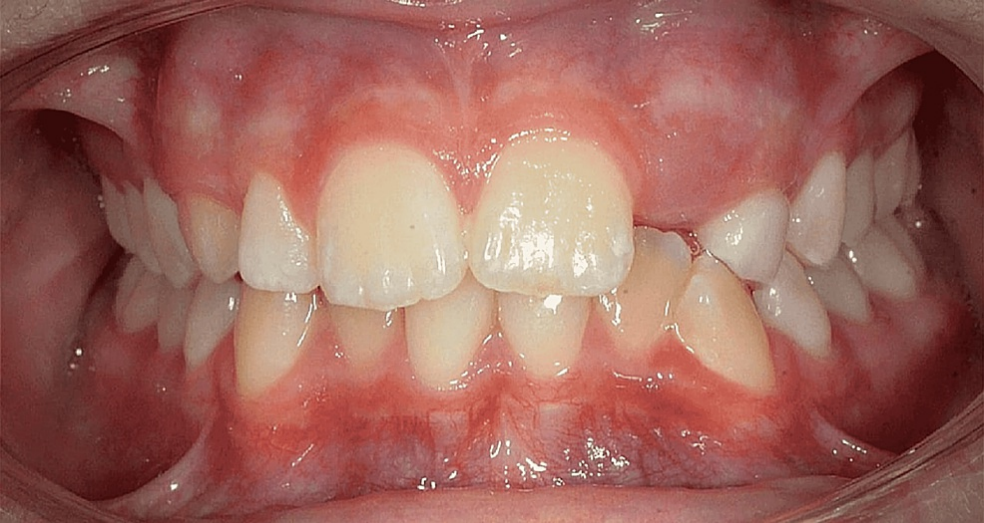
Frontal view photograph of a patient included in the study

The inclusion criteria were as follows: subjects with fully erupted teeth set except for third molars with the agenesis of unilateral/bilateral maxillary lateral incisors, and subjects with no tooth structure loss secondary to wasting diseases. The exclusion criteria were as follows: subjects with no pretreatment casts, mesiodistal restorations, and full teeth crown due to altered teeth dimensions (Figure 1).

Methods and analysis

**Figure 2:**
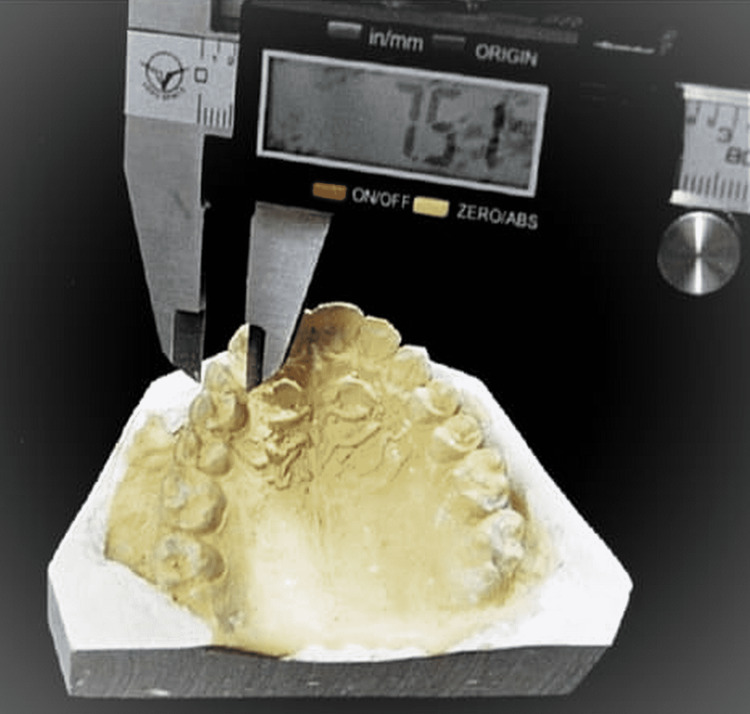
Vernier caliper measurements on the cast

All the dental cast measurements were done by a single expert in the field. The mesiodistal dimensions were measured and calculated for each tooth by using a digital caliper (Figure 2).

The values were rounded off to the nearest millimeter for each tooth. All the lateral incisors were excluded from the study owing to unilateral/bilateral missing lateral incisors. Left and right tooth sizes for both the dental arches were added, and the average was considered as the tooth size variable outcome.

The comparison of tooth sizes was done for the control and test groups. Further comparison was done for gender, inter arches, and tooth number. Statistical assessment of the data obtained was performed by using the analysis of variance (ANOVA) test and the SPSS Statistics software (IBM, Armonk, NY). A p-value <0.005 was considered statistically significant.

## Results

There were 19 (59.37%) unilateral missing lateral incisors and 13 bilateral missing lateral incisors. The age of the study subjects ranged from 11 to 42 years with a mean age of 15.78 years. For the purpose of comparison, 32 controls matched for age and sex were also included and compared against tests with missing lateral incisors. The demographic characteristics of the study subjects are listed in Table 1. The mean age of the study subjects in the test group was 15.89 ±6.95 years, and that in the control group was 15.87 ±6.54 years. There were 17 (53.12%) males and 15 (46.87%) females in the control as well as the test group.

**Table 1:** Demographic data of the study subjects SD: standard deviation

Characteristic	Subgroups		
Mean age, years, mean ±SD	Tests	15.89 ±6.95	
	Controls	15.87 ±6.54	
	Overall study group	15.78 ±6.85	
		%	N
Gender	Males (test)	53.12	17
	Females (test)	46.87	15
	Males (test)	53.12	17
	Females (test)	46.87	15
Missing maxillary lateral incisor (Test)	Unilateral	59.37	19
	Bilateral	40.62	13

The teeth dimensions were measured for all subjects in both test and control groups, and the results are summarized in Table 2.

**Table 2:** Tooth size dimensions in the test and control groups of study subjects SD: standard deviation

Tooth number	Groups	Males (n)	Dimension (mean ±SD)	P-value	Females (n)	Dimension (mean ±SD)	Group
36	Test	17	11.08 ±0.63	0.4770	15	10.78 ±0.58	0.2681
	Control	17	11.22 ±0.91		15	10.63 ±0.49	
35	Test	17	7.14 ±0.48	0.3262	15	7.06 ±0.43	0.7761
	Control	17	7.26 ±0.49		15	7.03 ±0.41	
34	Test	17	6.88 ±0.49	0.01	15	6.94 ±0.93	0.6314
	Control	17	7.20 ±0.50		15	7.03 ±0.50	
33	Test	17	6.63 ±0.59	0.02	15	6.40 ±0.30	0.1331
	Control	17	6.93 ±0.47		15	6.52 ±0.33	
32	Test	17	5.61 ±0.43	0.003	15	5.59 ±0.39	0.009
	Control	17	5.93 ±0.40		15	5.84 ±0.36	
31	Test	17	5.08 ±0.51	0.004	15	5.09 ±0.38	0.0888
	Control	17	5.41 ±0.36		15	5.25 ±0.36	
41	Test	17	5.19 ±0.49	0.07	15	5.06 ±0.40	0.0352
	Control	17	5.38 ±0.34		15	5.27 ±0.38	
42	Test	17	5.56 ±0.52	0.002	15	5.61 ±0.30	0.0138
	Control	17	5.94 ±0.45		15	5.82 ±0.36	
43	Test	17	6.66 ±0.64	0.14	15	6.35 ±0.43	0.0680
	Control	17	6.96 ±0.50		15	6.53 ±0.3	
44	Test	17	6.86 ±0.56	0.06	15	5.59 ±0.39	0.3638
	Control	17	7.13 ±0.52		15	5.84 ±0.36	
45	Test	17	7.10 ±0.48	0.252	15	7.04 ±0.51	0.4088
	Control	17	7.24 ±0.49		15	6.94 ±0.45	
46	Test	17	11.05 ±0.65	0.516	15	10.77 ±0.59	0.3374
	Control	17	11.18 ±0.92		15	10.64 ±0.48	
26	Test	17	9.83 ±0.78	<0.0001	15	9.90 ±0.58	0.8780
	Control	17	10.34 ±0.72		15	9.92 ±0.45	
25	Test	17	6.41 ±0.47	0.007	15	6.47 ±0.43	0.5222
	Control	17	6.71 ±0.39		15	6.54 ±0.44	
24	Test	17	6.75 ±0.58	0.006	15	6.83 ±0.43	0.5318
	Control	17	7.11 ±0.42		15	6.90 ±0.46	
23	Test	17	7.64 ±0.54	0.103	15	7.30 ±0.39	0.020
	Control	17	7.88 ±0.62		15	7.52 ±0.35	
22	Test	6	4.71 ±1.29	<0.0001	4	5.80 ±1.39	0.081
	Control	17	6.80 ±0.55		15	6.50 ±0.40	
21	Test	17	8.54 ±0.7	0.548	15	8.18 ±0.59	0.007
	Control	17	8.65 ±0.3		15	8.60 ±0.62	
11	Test	17	8.53 ±0.76	0.5909	15	8.25 ±0.67	0.421
	Control	17	8.63 ±0.72		15	8.58 ±0.60	
12	Test	4	5.81 ±0.95	<0.0001	5	5.21 ±0.88	<0.0001
	Control	17	6.77 ±0.62		15	6.53 ±0.41	
13	Test	17	7.77 ±0.53	0.0919	15	7.37 ±0.42	0.02
	Control	17	8.01 ±0.59		15	7.61 ±0.39	
14	Test	17	6.75 ±0.60	0.01	15	6.70 ±0.45	0.0327
	Control	17	7.08 ±0.37		15	6.93 ±0.39	
15	Test	17	6.50 ±0.39	0.009	15	6.47 ±0.42	0.5878
	Control	17	6.78 ±0.44		15	6.53 ±0.46	
16	Test	17	10.02 ±0.55	0.008	15	9.90 ±0.66	0.4170
	Control	17	10.47 ±0.76		15	10.01 ±0.38	

It was seen that premolars and molars were significantly smaller in the males of the test group in comparison to males in the control group, with a p-value of 0.01 for the lower first premolar. Significantly smaller maxillary lateral incisors were seen in the test group compared to the control group (p<0.0001 for both left and right sides). For the mandibular arch in males, significantly smaller left premolar, lateral incisor, central incisor, and left lateral incisors were seen with respective p-values of 0.01, 0.003, 0.004, and 0.002.

Among females, smaller teeth were seen in the maxillary arch anterior region. In the lower arch, left mandibular lateral incisor dimensions were significantly smaller in the test group compared to males (p=0.009). Except for the lateral incisor, differences in dimensions of other teeth were insignificant. With regard to maxillary arch in females, the left canine and central incisor were significantly smaller in the test group compared to controls with respective p-values of 0.020 and 0.007. On the right side, the lateral incisor and canine were significantly smaller in the test group with respective p-values of <0.0001 and 0.02 (Table 2).

It was observed that the lateral incisors of the maxillary arch were statistically smaller in the test group compared to the control group. This was seen in both males and females, with p-values of 0.001 and 0.0001 respectively. The same holds for the overall study group, with a p-value of <0.0001 (Table 3).

**Table 3:** Comparison of maxillary lateral incisors between the test and control groups of study subjects SD: standard deviation

Group	Tooth	Gender	Mean ±SD	P-value
Test	Upper lateral Incisors	Males	5.15 ±1.25	0.001
Control			6.79 ±0.58	
Test	Upper lateral Incisors	Females	5.56 ±1.20	0.0001
Control			6.51 ±0.40	
Test	Upper lateral Incisors	Combined	5.37 ±1.21	<0.0001
Control			6.64 ±0.51	

## Discussion

In this study, for comparative analysis, 32 age- and gender-matched controls were included and compared against tests with missing lateral incisors. The mean age of the study subjects in the test group was 15.89 ±6.95, and that in the control group was 15.87 ±6.54 years. There were 17 (53.12%) males and 15 (46.87%) females in the control as well as the test group. These demographics were comparable to those in the studies by Kokic [6] in 2004 and Uysal et al. [7] in 2005 where authors assessed similar characteristics.

Significantly smaller dimensions of molars and premolars were seen in the male subjects in the test group compared to the control group (p=0.01) in lower first premolar. Also, in the test group, significantly smaller maxillary lateral incisors were seen on both right and left sides (p<0.0001). In males, in the mandibular arches, left central incisors, lateral incisors, and premolars were seen with respective p-values of 0.004, 0.003, and 0.01. These findings are in agreement with the studies of Garib et al. [8] in 2010 and Baidas and Hashim [9] in 2005 where similar anterior and posterior teeth discrepancies were analyzed by authors.

In the maxillary anterior region, smaller teeth were seen, and in the lower arch, a significantly smaller lateral incisor was seen in the test group (p=0.009) compared to males. Other teeth had nonsignificant differences in terms of dimension, except for the lateral incisor. In females, in the maxillary arch, the left central incisor and canine had significantly lower dimensions in the test group compared to controls, with p-values of 0.007 and 0.02 respectively. Canine and lateral incisors had significantly lower dimensions in tests compared to controls, with p-values of 0.02 and <0.0001 respectively. These results were similar to those in the study by Rosa and Zachrisson [10] in 2010 where comparable findings were described by the authors.

The study results also showed that in the maxillary arch, lateral incisors had significantly lower dimensions in tests compared to controls in both females and males (p=0.001 for both). This was similar for both the study groups (p<0.0001). These findings were comparable to the results of Othman and Harradine [11] in 2007 and Peck et al. [12] in 2002.

There is scarce literature available on the topic of this study. Our study has a few limitations, such as the small sample size. Further studies with larger sample sizes are required to gain deeper insights into the topic.

## Conclusions

Within its limitations, the present study concludes that agenesis of maxillary lateral incisors in males leads to smaller posterior teeth bilaterally. In females, this agenesis leads to smaller anterior teeth in the maxilla. Irrespective of gender, maxillary lateral incisor agenesis leads to the formation of smaller and peg-shaped laterals. These findings confirm the role of maxillary lateral incisor agenesis in malocclusion development. Our study has a few limitations, mainly the small sample size and the single-institution setting. Hence, more long-term studies involving a larger number of casts are required to reach definitive conclusions about the topic.
